# Preventing and Mitigating SARS-CoV-2 Transmission — Four Overnight Camps, Maine, June–August 2020

**DOI:** 10.15585/mmwr.mm6935e1

**Published:** 2020-09-04

**Authors:** Laura L. Blaisdell, Wendy Cohn, Jeff R. Pavell, Dana S. Rubin, Jeffrey E. Vergales

**Affiliations:** ^1^Center for Outcomes Research and Evaluation, Department of Pediatrics, Maine Medical Center Research Institute and Maine Medical Center, Portland, Maine; ^2^Department of Public Health Sciences, University of Virginia School of Medicine, Charlottesville, Virginia; ^3^Englewood Health, Inc., Englewood, New Jersey; ^4^Department of Pediatrics and Psychiatry, Boston University School of Medicine, Boston, Massachusetts; ^5^Department of Pediatrics, University of Virginia School of Medicine, Charlottesville, Virginia.

The World Health Organization declared coronavirus disease 2019 (COVID-19) a pandemic on March 11, 2020.* Shortly thereafter, closures of 124,000 U.S. public and private schools affected at least 55.1 million students through the end of the 2019–20 school year.^†^ During the summer of 2020, approximately 82% of 8,947 U.S. overnight camps did not operate.^§^ In Maine, only approximately 20% of 100 overnight camps opened.^¶^ An overnight camp in Georgia recently reported SARS-CoV-2, the virus that causes COVID-19, transmission among campers and staff members when nonpharmaceutical interventions (NPIs) were not strictly followed (*1*); however, NPIs have been successfully used to mitigate SARS-CoV-2 transmission among military basic trainees (*2*). During June–August 2020, four overnight camps in Maine implemented several NPIs to prevent and mitigate the transmission of SARS-CoV-2, including prearrival quarantine, pre- and postarrival testing and symptom screening, cohorting, use of face coverings, physical distancing, enhanced hygiene measures, cleaning and disinfecting, and maximal outdoor programming. During the camp sessions, testing and symptom screening enabled early and rapid identification and isolation of attendees with COVID-19. Among the 1,022 attendees (staff members and campers) from 41 states, one territory, and six international locations, 1,010 were tested before arrival; 12 attendees who had completed a period of isolation after receiving a diagnosis of COVID-19 2 months before arrival were not tested. Four (0.4%) asymptomatic attendees received positive SARS-CoV-2 test results before arrival; these persons delayed their arrival, completed 10 days of isolation at home, remained asymptomatic, and did not receive any further testing before arrival or for the duration of camp attendance. Approximately 1 week after camp arrival, all 1,006 attendees without a previous diagnosis of COVID-19 were tested, and three asymptomatic cases were identified. Following isolation of these persons and quarantine of their contacts, no secondary transmission of SARS-CoV-2 occurred. These findings can inform similar multilayered public health strategies to prevent and mitigate the introduction and transmission of SARS-CoV-2 among children, adolescents, and adults in congregate settings, such as overnight camps, residential schools, and colleges.

Summer camps are a $26 billion dollar industry; approximately 15,000 day and overnight camps in the United States employ approximately 1.5 million staff members and host an estimated 26 million children annually. The Maine Department of Health and Human Services (DHHS) licenses Maine summer camps, which serve 20,000–25,000 children from the United States and other countries each year. Previous studies suggest that isolation and physical distancing measures likely mitigated disease during the influenza pandemic of 1918 and prevented spread of the coronavirus SARS-CoV, which caused the severe acute respiratory syndrome (SARS) epidemic in 2003 (*3*,*4*). During the 2009 influenza A virus (pH1N1) pandemic, CDC issued guidance for influenza prevention and control in camp settings focusing on early identification and isolation of ill persons and enhanced hygiene.** Camps operating in Maine during the pH1N1 2009 season followed public health guidance and implemented recommended preventive measures. Although many camps reported influenza-like illness and outbreaks, major disruptions were not reported (*5*).

To prevent, identify, and mitigate spread of COVID-19, four Maine overnight summer camps with similar size, session duration, and camper and staff member characteristics opened with uniform NPIs, including precamp quarantine, pre- and postarrival testing and symptom screening, cohorting, and physical distancing between cohorts. In addition, camps required use of face coverings, enhanced hygiene measures, enhanced cleaning and disinfecting, maximal outdoor programming, and early and rapid identification of infection and isolation.

All attendees were instructed to quarantine with their family unit (unless parents were essential workers^††^) for 10–14 days before camp arrival. No camp restricted attendance from any part of the country or globally but did advise on mode of travel (preferred mode was direct to camp in family vehicle; riders on camp buses wore face coverings, with physical distancing monitored by staff members; and air travelers were instructed to wear face coverings while traveling). Study activities were conducted by the medical directors and health staff members at each camp and under exempt approval by the Institutional Review Board of the University of Virginia.

Attendees with COVID-19 were defined as detection of SARS-CoV-2 by reverse transcription–polymerase chain reaction (RT-PCR) testing. Approximately 5–7 days (mean = 2.4–9.4 days) before camp arrival, 1,010 of the 1,022 attendees were tested for SARS-CoV-2 by RT-PCR at the attendees’ primary care providers or at commercial laboratories that provided services directly to consumers, including camps and schools according to Food and Drug Administration’s Emergency Use Authorizations. Attendees with self-reported symptoms consistent with COVID-19 as defined by CDC (https://www.cdc.gov/coronavirus/2019-ncov/symptoms-testing/symptoms.html) before camp arrival were referred to their primary care provider for further evaluation. Three of four camps mandated submission of test results before camp entry, and delays in receipt of test results caused one camp to isolate 15 campers until negative results were known, up to 4 days after camp arrival.

To address potential late exposures or exposures during travel, all camps quarantined attendees by cohort for 14 days after camp arrival, regardless of testing or screening results. Each camp implemented NPIs with careful attention to the population served, physical attributes of the camp, and camp-specific daily programming to identify and mitigate high-transmission–risk activities occurring between cohorts. All attendees received instruction on hygiene measures such as cough and sneeze etiquette and hand hygiene, with the requirement to clean hands with soap and water or hand sanitizer containing a minimum of 60% ethanol or 70% isopropanol before and after all activity periods, meals, and other high-touch interactions. Compliance with all NPIs was monitored by staff members. Staff members did not leave camp during the session for days off. 

After camp arrival, campers and staff members were screened by health staff members at least daily (at one camp twice daily) for fever (temperature >100.4°F [38°C]) with infrared thermometers and through direct questioning for symptoms consistent with COVID-19. Programmatic changes to usual camp activities included limiting indoor activities that mixed cohorts, staggering dining periods or dining outdoors, cohort-specific programming, and limiting sports to those that allowed for physical distancing between staff members and cohorts. Stable cohorts were based on living quarters (e.g., bunk assignment) or age division and ranged in number from 5–44 attendees. If interacting outside the cohort, attendees were required to wear face coverings and maintain a physical distance of 6 feet for a minimum of 14 days. Bathroom use was organized by cohort using separate bathrooms or staggering use. In general, cleaning and disinfection of the camps followed the Maine Center for Disease Control and American Camp Association Field Guide for Camps on Implementation of CDC Guidance.^§§^ Shared items were cleaned and disinfected as much as possible, with high touch areas (e.g., door handles or railings) being cleaned more frequently. Personal sports equipment and shared items were disinfected immediately after use, or a minimum of 24 hours was required before subsequent use. Kitchens followed standard protocols, as well as state COVID-19 protocols for restaurants. Bathrooms were cleaned and disinfected twice daily. Camps attempted to use single-use items, such as milk cartons and single-use condiment packs or silverware, to the extent possible.

RT-PCR testing was repeated a mean of 4.1 to 9.1 days after camp arrival for 1,006 attendees, with results available approximately 2–3 days later; no attendees declined testing. Attendees with positive SARS-CoV-2 test results or those who reported symptoms consistent with COVID-19 were isolated immediately, and their cohort was quarantined until the attendee received a negative test result.

Before the 1,022 attendees departed for camp, four (0.4%) asymptomatic attendees received positive SARS-CoV-2 test results and delayed their arrival; they were subsequently isolated for 10 days at their homes, were not retested before camp entry, were considered to not have COVID-19 at time of camp arrival, and did not receive any further testing for the duration of their attendance. Twelve attendees (nine staff members and three campers) were not tested before travel to camp because they had completed a period of isolation after experiencing symptoms and having received positive SARS-CoV-2 RT-PCR test results in the 2 months before camp opening. The remaining 1,006 attendees received negative SARS-CoV-2 test results. 

During June–August, the combined attendance of the four camps included 642 children and 380 staff members, aged 7–70 years, from 41 states with a variety of 7-day average rate of SARS-CoV-2 infection (Figure); 1.8% of camp attendees^¶¶^ (10 staff members and eight campers) came from six international locations (Bermuda, Canada, Mexico, South Africa, Spain, and United Kingdom) and Puerto Rico (Table 1). Camp sessions ranged from 44 to 62 days (including a 14-day staff member orientation) during June 15–August 16, 2020. The number of campers in cabins (including dormitory-style quarters) ranged from five to 44 campers (Table 2). No attendee reported a condition that precluded wearing a face covering, and all attendees were observed to comply with use of face coverings and physical distancing.

**FIGURE:**
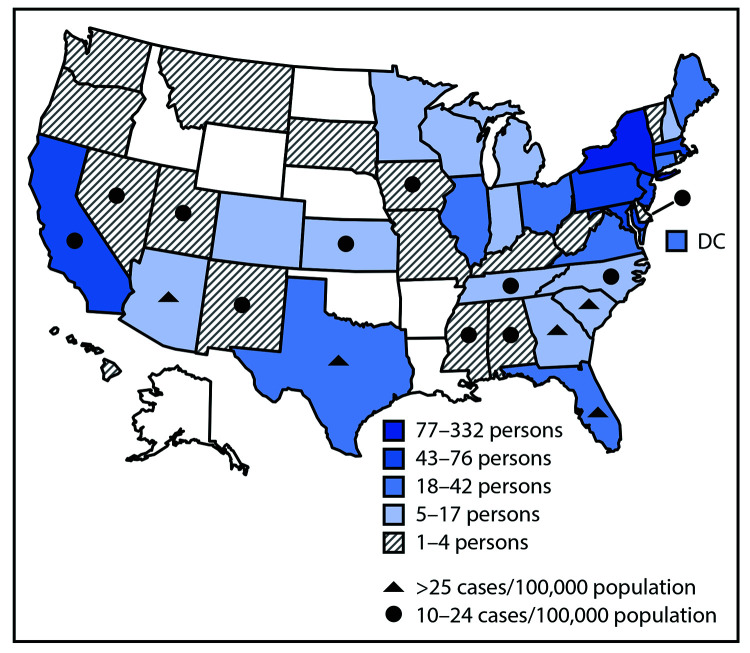
Camp population, by home state* and by 7-day daily average rate of SARS-CoV-2 infection^†^ in home state as calculated on July 1, 2020^§^ — four overnight camps, Maine, June–August 2020 * Combined attendance by quintiles of home state of the four camps included 642 children and 380 staff members aged 7–70 years, representing 41 states; 18 attendees (10 staff members and eight campers) originating from six international locations (Bermuda, Canada, Mexico, South Africa, Spain, and United Kingdom) and Puerto Rico are not shown on the map. States with incidence <10 cases per 100,000 population not designated. Jenks natural breaks used for attendee classification by home state. ^†^ Average case rate indexed to the state-specific population sourced by Harvard Global Health Institute (https://globalepidemics.org). ^§^ July 1, 2020, is when the state of Maine allowed overnight camps to open for business.

**TABLE 1:** Characteristics of campers and staff members,* — four overnight camps, Maine, June–August 2020

Characteristic	No. (%)*
**Total**	**1,022 (100)**
**Sex**	
Male	470 (46)
Female	552 (54)
**Role**	
Camper	642 (63)
Staff member	380 (37)
**Age group, yrs^†^**	
7–8	30 (3)
9–10	135 (13)
11–12	175 (17)
13–14	184 (18)
15–18	133 (13)
19–21	151 (15)
22–29	126 (12)
30–49	45 (4)
50–70	43 (4)
**Home region^§^**	
Middle Atlantic	438 (43)
South	187 (18)
New England	173 (17)
Midwest	105 (10)
West Coast	100 (10)
International^¶^	18 (2)

*Percentages might not sum to 100% because of rounding.

^†^ Age was ascertained at time of camp entry.

^§^ Domestic home regions defined according to U.S. Census regions: *New England*: Connecticut, Maine, Massachusetts, New Hampshire, Rhode Island, and Vermont. *Middle Atlantic*: New Jersey, New York, Pennsylvania. *Midwest*: Illinois, Indiana, Iowa, Kansas, Michigan, Minnesota, Missouri, Nebraska, North Dakota, Ohio, South Dakota, and Wisconsin. *South*: Alabama, Arkansas, Delaware, District of Columbia, Florida, Georgia, Kentucky, Louisiana, Maryland, Mississippi, North Carolina, Oklahoma, South Carolina, Tennessee, Texas, Virginia, and West Virginia. *West*: Alaska, Arizona, California, Colorado, Hawaii, Idaho, Montana, Nevada, New Mexico, Oregon, Utah, Washington, and Wyoming.

^¶^ International included six international locations (Bermuda, Canada, Mexico, South Africa, Spain, and United Kingdom) and Puerto Rico.

**TABLE 2:** Camp session dates,* number of camp days, median cabin population, and enrollment, by camp — four overnight camps, Maine, June–August 2020

**Characteristic**	**Camp A**	**Camp B**	**Camp C**	**Camp D**
**Camp session dates**	Jun 25–Aug 8, 2020	Jun 25–Aug 8, 2020	Jun 15–Aug 18, 2020	Jun 23–Aug 9, 2020
**Total camp days**	44	44	62	47
**Median 2020 cabin population (range)^†^**	7 (7–10)	12 (5–44)	5 (5–25)	8 (5–30)
**Total 2020 enrollment**	276	287	202	257
Campers (n = 642)	156	180	140	166
Staff members (n = 380)	120	107	62	91
**Total usual enrollment^§^**	380	400	240	327
Campers	250	230	155	200
Staff members	130	170	85	127
**Percentage of usual enrollment, %^§^**	72.6	71.8	84.2	78.6

*Camp sessions inclusive of additional 14-day staff member orientation.

^†^ Includes dormitory style quarters with common living areas.

^§^ Usual enrollment was defined as normal capacity of each camp during 2017–2019.

Daily symptom checks identified 12 attendees (one staff member and 11 campers) (1.2%) with signs or symptoms compatible with COVID-19; symptomatic persons were immediately isolated and tested, and their cohorts were quarantined until test results were available. All 12 isolated attendees received negative test results, after which isolation and cohort quarantine were discontinued.

Three asymptomatic attendees at three different camps (two staff members and one camper) (0.3%) received positive SARS-CoV-2 test results after arrival at camp and were rapidly isolated and their cohorts (sized five, six, and 30 attendees) quarantined for 14 days per state and CDC guidance. Both asymptomatic staff members isolated for 10 days and received negative test results twice 24 hours apart at the end of their isolation. The asymptomatic camper was isolated on day 3 after testing when positive test results were received. The camper was retested on days 4 and 5 after a positive test result and released from isolation on day 8 after a second negative result was received (per CDC isolation termination guidelines at that time). The 30 members of the camper’s cohort were retested on days 3 and 4 after the asymptomatic camper’s initial positive test result. No cohort members received a positive test result, and all were released from quarantine on day 8 after the asymptomatic camper’s positive test result. No secondary transmission was identified.

## Discussion

Diligent use of multiple NPIs was successful in preventing and mitigating SARS-CoV-2 transmission in four Maine overnight camps. Although no single intervention can prevent SARS-CoV-2 transmission, a multilayered use of NPIs allowed camps to prevent transmission and quickly identify campers or staff members with SARS-CoV-2 infection to successfully mitigate spread. Camps did not rely on testing as a sole NPI. Notably, stable, small, segregated cohorts allowed camps to isolate and quarantine a wide age range of younger attendees with potential COVID-19 symptoms and exposures while continuing camp operations in other cohorts.

Testing and quarantine before staff member and camper arrival was essential to identifying SARS-CoV-2 infection and preventing introduction of virus into these congregate settings of younger adults who might be only mildly symptomatic or presymptomatic (*6*–*9*). Prearrival testing with timely results, strict quarantining, and NPI use during transit were important, as was conscientious NPI use in the first 2 weeks after arrival. Testing after camp arrival identified three asymptomatic attendees with positive SARS-CoV-2 RT-PCR test results, but because these attendees were isolated and their cohorts quarantined, no transmission in the congregate setting or cohort occurred. Screening for symptoms after camp arrival identified 12 attendees who were isolated, and their cohorts were quarantined while awaiting test results. Both isolated and quarantined groups returned to the general camp population after the symptomatic attendees received negative SARS-CoV-2 test results.

The findings in this report are subject to at least five limitations. First, the degree of adherence to NPIs was not measured. Second, not testing all campers and staff members at the end of sessions might have missed asymptomatic transmission. Third, all camps were single sessions and interventions might not have similar results in multiple session overnight camps. Fourth, travel was assumed to be from home state as documented but intermediate travel might have occurred and attendees might not possess the same risk as other persons in their state. Finally, the low rate of COVID-19 in this study increases the likelihood that NPIs would be effective for at least some duration.

These findings demonstrate that multilayered public health prevention and mitigation strategies in an overnight camp setting can identify and prevent SARS-CoV-2 transmission, regardless of the prevalence of SARS-CoV-2 transmission in the domestic and international communities from which campers and staff members are arriving. Prearrival quarantine and testing, access to timely test results, cohorting, and the ability to isolate and quarantine during camp allowed prevention and early identification of infection that might not be practicable or feasible in all settings. These findings have important implications for the successful implementation of COVID-19 mitigation strategies in other overnight camps, residential schools, and colleges.

**Summary**

**What is already known about this topic?**

Nonpharmaceutical interventions (NPIs) have been shown to decrease spread of communicable disease. Data on the effectiveness of NPIs on the prevention and mitigation of SARS-CoV-2 transmission among children and adolescents in congregate settings are limited.

**What is added by this report?**

During the 2020 summer camp season, four Maine overnight camps with 1,022 attendees from 41 states and international locations implemented a multilayered prevention and mitigation strategy that was successful in identifying and isolating three asymptomatic COVID-19 cases and preventing secondary transmission.

**What are the implications for public health practice?**

Understanding successful interventions to prevent and mitigate SARS-CoV-2 transmission in overnight camps has important implications for similar congregate settings such as day camps and schools with the same age range.

## Footnotes

* https://www.who.int/dg/speeches/detail/who-director-general-s-opening-remarks-at-the-media-briefing-on-covid-19---11-march-2020.

† https://www.edweek.org/ew/section/multimedia/map-coronavirus-and-school-closures.html.

§ https://www.acacamps.org/press-room/aca-facts-trends.

¶
https://mainecamps.org .

** https://www.cdc.gov/h1n1flu/camp.htm.

†† Families of essential workers were instructed to limit interaction with camper to the degree possible in the 10–14 days leading up to camp.

§§
https://www.acacamps.org/resource-library/coronavirus/camp-business/camp-operations-guide-summer-2020 .

¶¶ Camps were for children aged 8–15 years. Staff members are aged >15 years.
